# Cavity-Induced Optical Nonreciprocity Based on Degenerate Two-Level Atoms

**DOI:** 10.3390/nano14151236

**Published:** 2024-07-23

**Authors:** Chuan-Zhao Qi, Jia-Rui Zheng, Yuan-Hang Tong, Ruo-Nan Li, Dan Wang, Liang-Hui Huang, Hai-Tao Zhou

**Affiliations:** 1 Sanli Honors College, Shanxi University, Taiyuan 030006, China; 202109031048@email.sxu.edu.cn (C.-Z.Q.); 202102301238@email.sxu.edu.cn (J.-R.Z.); 202101304629@email.sxu.edu.cn (Y.-H.T.); 2 State Key Laboratory of Quantum Optics and Quantum Optics Devices, Institute of Opto-Electronics, School of Physics and Electronic Engineering, Shanxi University, Taiyuan 030006, China; 202222607023@email.sxu.edu.cn (R.-N.L.); huanglh06@sxu.edu.cn (L.-H.H.); 3 Collaborative Innovation Center of Extreme Optics, Shanxi University, Taiyuan 030006, China

**Keywords:** optical nonreciprocity, single-dark-state peak, degenerate two-level system, strong coupling

## Abstract

We developed and experimentally realized a scheme of optical nonreciprocity (ONR) by using degenerate two-level atoms embedded in an optical ring cavity. For the degenerate transition F_g_ = 4 ↔ F_e_ = 3, we first studied the cavity-transmission property in different coupling field configurations and verified that under the strong-coupling regime, the single-dark-state peak formed by electromagnetically induced transparency (EIT) showed ONR. The stable ground-state Zeeman coherence for Λ-chains involved in the degenerate two-level system was found to be important in the formation of intracavity EIT. However, different from the three-level atom–cavity system, in the degenerate two-level system, the ONR effect based on intracavity EIT occurred only at a low probe intensity, because the cavity–atom coupling strength was weakened in the counter-propagating probe and coupling field configuration. Furthermore, ONR transmission with a high contrast and linewidth-narrowing was experimentally demonstrated.

## 1. Introduction

Optical nonreciprocity (ONR) has attracted widespread interest due to its important applications in optical communications and optical information processing [1,2,3]. Traditionally, ONR has been achieved based on the magneto-optical effect [4], which poses challenges in miniaturization and integration. Thus, the exploration of magnet-free ONR avenues has emerged in recent years. Significant advancements have been made by using methods such as nonlinear optics [5,6,7], optomechanical interactions [8,9,10], chiral quantum optics [11,12,13], tunable photonic crystals [14,15], cold atomic Bragg lattices [16,17], and hot atoms [18,19,20,21,22].

One of the most commonly used methods to achieve ONR is thermal motion. For instance, considering an ensemble of Λ-type three-level hot atoms, when the probe and coupling beams co-propagate in the atoms, the electromagnetically induced transparency (EIT) effect occurs (two-photon resonance is achieved because the Doppler shift is of equal magnitude for the co-propagating coupling and probe field, i.e., Doppler-free) [23]. However, if the probe beam counter-propagates with respect to the coupling beam, the strong absorption of the hot atoms hinders the transmission of the probe beam due to the Doppler shift. This chiral response of atoms dependent on a unidirectional coupling field breaks the system’s time-reversal symmetry and enables the ONR transmission of the probe light [18]. Furthermore, when combining this ensemble with an optical cavity, the enhancement in atomic nonlinearity can boost ONR efficiency and sensitivity [21,22], which has facilitated the development of quantum devices, such as optical isolators [24,25], optical switches and routers [26,27,28,29,30], and all-optical logic gates [31,32,33].

In this study, we experimentally demonstrated a scheme for achieving ONR with a degenerate two-level atom–cavity system. For the degenerate transition F_g_ ↔ F_e_ with the ground-state angular momentum (F_g_) being larger than the excited-state angular momentum (F_e_), we first studied the transmission of the probe beam in free space. It was found that ONR based on free-space EIT is not perfect due to frequency degeneracy. By embedding the hot degenerate atoms into a ring cavity, however, ONR cavity transmission can be achieved at low probe intensity, which benefits from the strong-coupling property of the atom–cavity system. By combining all Zeeman sublevels of the atoms and the strong-coupling characteristics of the atom–cavity system, we qualitatively analyzed the experimental results. Finally, by changing the parameters of the coupling field, ONR with high contrast and a wide frequency-tuning range was experimentally investigated.

## 2. Experimental Setup and Results

**Figure 1:**
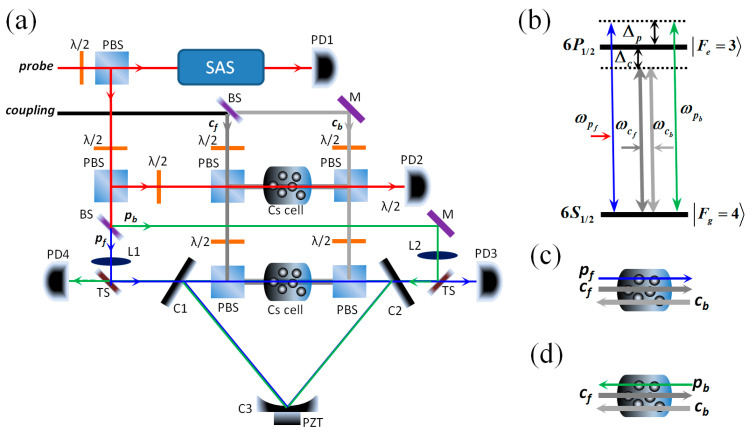
(**a**) Schematic of experimental setup. (**b**) Diagram of atom energy levels for the atoms. Schematic showing only p_f_ (**c**) or only p_b_ (**d**) seeded into the atom–cavity system.

The schematic diagram of our experimental setup is shown in Figure 1a. Two independent extended cavity diode lasers (ECDLs) with a wavelength of 894.5 nm were used as probe and coupling lasers. In our experimental setup, we defined the direction in which light traveled from left to right as forward and the opposite direction as backward. The probe laser was divided into three parts with two combinations of a half-wave plate (λ/2) and a polarization beam splitter (PBS): one part was used for the saturation absorption spectrum (SAS) and was detected with a photo detector (PD1); one part was used for the probe light of the free-space EIT system and was detected with PD2; and one part was further split into two parts (denoted by p_f_ and p_b_) with a 50/50 beam splitter (BS). We used a three-mirror ring cavity consisting of two plane mirrors, C1 and C2, with the same transmissivity, 3%, and a plano-concave super mirror, C3, with a radius of curvature of 1000 mm and a reflectivity of 99.99%, mounted on a piezoelectric transducer (PZT) for cavity frequency scanning and locking. The single cavity length (L) was about 530 mm. A 75 mm long intracavity Cs cell with anti-reflection-coated end windows was wrapped with µ-metal sheets for magnetic field shielding and heat tape for temperature control. Probe light p_f_ (p_b_), with horizontal polarization, was reflected by a beam splitter with a transmissivity of 90% (TS) and then injected into C1 (C2) along the forward (backward) direction; it circulated in the atom–cavity system as cavity mode, and its output was detected with PD3 (PD4). The coupling laser was split into two parts (denoted by c_f_ and c_b_) with a BS. Coupling light c_f_ (c_b_), with vertical polarization, was reflected by a PBS and passed through the Cs cell along the forward (backward) direction. Notably, the two counter-propagating coupling beams, which were collinear with the probe cavity mode, formed a standing wave (SW) field in the intracavity Cs cell and were then reflected out of the cavity by two PBSs to avoid circulation in the cavity. At the center of the cavity, the effective diameters of the probe and coupling light were about 380 μm and 575 μm, respectively. The finesse of the ring cavity was degraded from 100 to 36 because of the linear loss of the inserted Cs cell and the two PBSs. The temperature of the Cs cell both in the free space and in the cavity was controlled at 40 °C.

The diagrams of the energy levels of the atoms are shown in Figure 1b. Our experiment was performed in the D1 line of ^133^Cs atoms. The probe and coupling lasers drove the same transition: |6^2^S_1/2_, F_g_ = 4⟩ ↔ |6^2^P_1/2_, F_e_ = 3⟩. The coupling laser was frequency-locked to the nearby transition frequency, ω_ge_, with detuning Δ_c_ (Δ_c_ = ω_c_ − ω_ge_), and the probe laser was frequency-scanned across ω_ge_ with detuning Δ_p_ (Δ_p_ = ω_p_ − ω_ge_). Figure 1c and 1d show the interaction process of the atom–cavity system when only p_f_ or only p_b_ was injected in the intracavity Cs cell, respectively. We define Δ_q_ = ω_q_ − ω_ge_ as the detuning of the qth probe cavity mode from the atomic resonance frequency.

**Figure 2:**
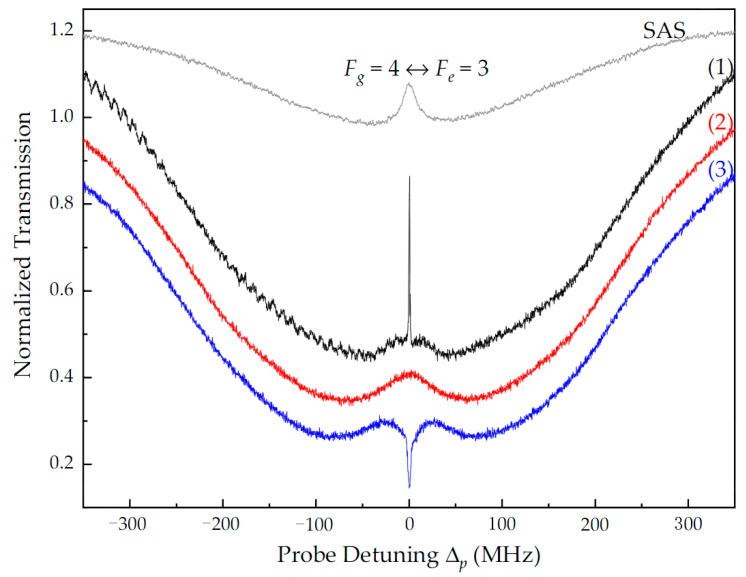
Transmission spectra of probe light for different configurations of the coupling fields in free space: (1) only c_f_ with power P_cf_ = 1 mW, (2) only c_b_ with P_cb_ =1 mW, and (3) SW c_s_ with P_cf_ = P_cb_ = P_cs_ = 1 mW. The other experimental parameters are P_p_ = 10 μW and Δ_c_ = 0.

In Figure 2, we compare the transmission spectra of the probe light for different configurations of the coupling fields in free space, as detected with PD2 (see Figure 1a). The gray curve is the SAS of the probe laser, which was used to search for the atomic transition and mark the frequency detuning of the coupled laser. When only the co-propagating coupling field c_f_ was injected into the Cs cell, a narrow transparency peak with a linewidth of ~1.2 MHz was obtained because two-photon resonance was achieved (that is, there was no Doppler effect, Δ_p_ = Δ_c_), i.e., the so-called EIT effect [23], as represented by the black curve (1) in Figure 2. Predictably, when only the counter-propagating field c_b_ was injected, the EIT peak disappeared due to the Doppler shift of the hot atom, as shown by the red curve (2) in Figure 2. However, transparency with linewidth broadening appeared near the center of two-photon resonance. This is because the frequency degenerate coupling and probe light excited atoms in the same ground state, most of which were excited by the stronger coupling light, and eventually the absorption of the probe light by the atoms was weakened. Notably, this was independent of the propagating direction of the coupling light. Therefore, there was still a weak probe light that could pass through the Cs cell, which led to imperfect ONR. The SW field c_s_ formed when c_f_ and c_b_ were simultaneously injected, which caused periodic modulation of the refractive index of the atom, accompanied by anomalous dispersion and strong absorption of the probe light [34]. Therefore, a strong absorption dip appeared at the center of two photon resonance, as shown by the blue curve (3) in Figure 2, which is the so-called electromagnetically induced absorption (EIA) [35]. Here, the probe light could not pass through the Cs cell, whether it propagated along the forward or backward direction. 

**Figure 3:**
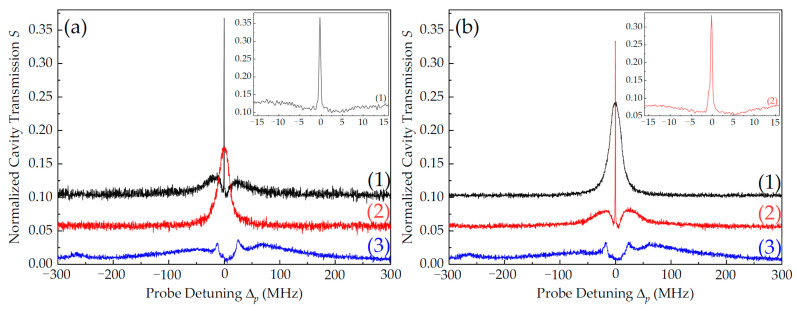
Normalized cavity transmission spectra of the forward probe light, p_f_ (**a**), and backward probe light, p_b_ (**b**), in different coupling light configurations: (1) only c_f_ with P_cf_ = 20 mW, (2) only c_b_ with P_cb_ = 20 mW, and (3) c_s_ with P_cs_ = 20 mW. The other experimental parameters are P_pf_ = P_pb_ = 1.8 mW, T = 40 °C, and Δ_q_ = Δ_c_ = 0.

Inspired by the ONR in three-level atom–cavity systems [18,22], we placed the degenerate two-level atoms in a three-mirror ring cavity (see Figure 1). Figure 3 plots the normalized cavity transmission spectra, S, of probe light at Δ_q_ = Δ_c_ = 0 (to facilitate discrimination, we shifted the different curves in the same figure up at equal intervals). Here, S = V_out_/V_pf(b)_, where V_pf(b)_ is the intensity of the forward (backward) probe light injected into the cavity and V_out_ is the intensity of the probe light output from the cavity. The values of corresponding V were obtained by the photo detectors with the same magnification. For the forward probe light, p_f_ (Figure 1c), when only the forward coupling light, c_f_, was injected (P_cf_ = 20 mW), apart from two vacuum Rabi splitting (VRS) peaks which resulted from the strong coupling between atoms and the cavity [36], a narrowed single-dark-state peak (intracavity EIT) was observed at atomic resonance (Δ_p_ = 0), as shown by the black line (1) in Figure 3a. Compared with the EIT linewidth of 1.2 MHz in free space, this intracavity EIT linewidth was narrow halved (~0.6 MHz), as shown in the illustration in Figure 3a. When only the backward coupling light, c_b_, was injected with P_cb_ = 20 mW, a widened cavity mode without VRS appeared at atomic resonance, as shown by the red line (2) in Figure 3a. For the backward probe light, p_b_ (Figure 1d), the results are opposite, as shown by the lines (1) and (2) in Figure 3b. In the configuration of the SW coupling field (P_cs_ = 20 mW), the cavity transmission spectra were similar for both p_f_ and p_b_; the original single-dark-state peak at atomic resonance transformed into symmetrical double-dark peaks on both sides of the atomic resonance because of EIA, as shown by the blue lines (3) in Figure 3a,b. It is worth emphasizing that the double-dark-state peaks exhibited optical reciprocity (OR) due to the symmetry of the system, but the transmission efficiencies were much lower than that of the single-dark-state peak because the double-dark-state peaks were not resonant with the cavity mode (Δ_q_ = 0). 

As mentioned above, when probe light counter-propagates with respect to the coupling light, cavity transmission shows imperfect ONR (or OR) at atomic resonance, which is different from what is observed in the Λ-type three-level atom–cavity system [18]. The reason is that in our experiment, the counter-propagating coupling light excited most of the degenerate ground-state atomic population, which reduced the absorption of the probe cavity mode by the intracavity atoms and restrained the strong-coupling efficiency of the atom–cavity system. By decreasing the input power of the probe light, the strong-coupling effect of the atom–cavity system could be obtained. 

**Figure 4:**
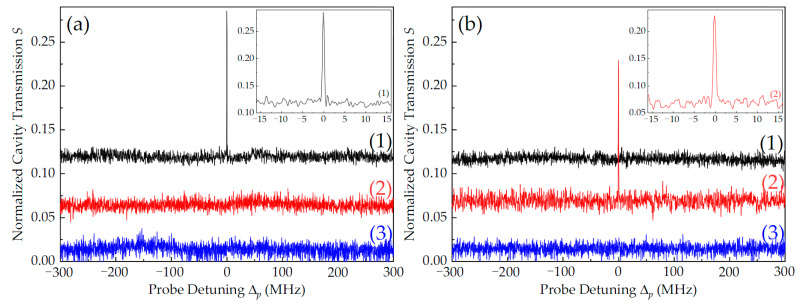
Normalized cavity transmission spectra of forward probe light p_f_ (**a**) and backward probe light p_b_ (**b**) with optical power of P_pf_ = P_pb_ = 0.4 mW. The other experimental parameters are the same as those in Figure 3.

Figure 4 shows the cavity transmission at P_pf_ = P_pb_ = 0.4 mW. When the probe and coupling light co-propagated, a linewidth-narrowed intracavity EIT peak could be obtained at Δ_p_ = Δ_c_ = 0, as shown by the black line (1) in Figure 4a or the red line (2) in Figure 4b. When the probe and coupling light counter-propagated, the cavity mode at atomic resonance disappeared due to the Doppler shift of the atoms and the strong-coupling effect of the atom–cavity system, as shown by the red line (2) in Figure 4a or the black line (1) in Figure 4b. Here, ONR based on degenerate two-level intracavity EIT was obtained. We define the contrast of ONR as η = (S_co_ − S_coun_)/(S_co_ + S_coun_) [14], where S_co(coun)_ is the normalized cavity transmission of the probe light that co-propagates (counter-propagates) with respect to the coupling light at Δ_p_ = Δ_c_ = 0. The contrast, η, reached nearly 90% for both p_f_ and p_b_; see the illustration in Figure 4. Compared with the results in Figure 3, the VRS peaks were very weak to almost invisible because of the large absorption and linear loss of the atom–cavity system in the case of the low-power probe light, and the contrast of ONR was excellent. In addition, the double-dark-state peaks generated by the SW coupling field were also suppressed, as shown by the blue lines (3) in Figure 4.

## 3. Theoretical Analysis and Further Experimental Investigation

**Figure 5:**
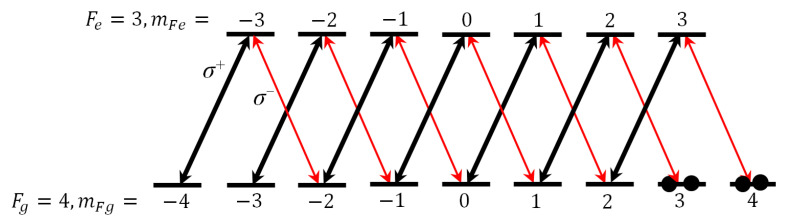
Zeeman sublevels for the degenerate transitions F_g_ = 4 ↔ F_e_ = 3. The black arrow represents the σ^+^ component of the coupling light, and the red arrow represents the σ^−^ component of the probe light.

Here, taking into account all Zeeman sublevels of the degenerate two-level configuration for transition F_g_ = 4 ↔ F_e_ = 3, we provide a theoretical analysis of the experimental results. As is known, a linearly polarized beam consists of left-handed (σ^+^) and right-handed (σ^−^) circularly polarized components which act on the corresponding σ transition. In our experiment, the coupling and probe beams were both linearly polarized and perpendicular to each other. For co-propagating coupling and probe lights, due to the absence of the Doppler effect, the σ^+^ (σ^−^) component of the former and the σ^−^(σ^+^) component of the latter can form coherent population trapping (CPT) between ground Zeeman sublevels |m_Fg_⟩ and |m_Fg_ ± 2⟩ and then establish two stable Λ-type EIT chains [37], as shown in Figure 5. Therefore, the transmission of probe light in free space can be regarded as the superposition of multiple Λ-type EIT configurations, as it shows a narrow transparency peak at two-photon resonance (see the black curve in Figure 2). Furthermore, the transparency peak of probe light output from the cavity (i.e., the intracavity EIT peak) is narrowed again (see Figure 3 and Figure 4).

Based on the above consideration, the degenerate two-level transition F_g_ = 4 ↔ F_e_ = 3 can be simplified as a multiple Λ-type three-level system. Therefore, the physical mechanism of the formation of single-/double-dark-state peaks in a degenerate two-level atom–cavity system can be explained by using the semi-classical theory of three-level atoms driven by a dichromatic coupling field. Taking the forward probe light as an example, p_f_ drives the transition |F_g_ = 4, m_F_ = 4⟩ ↔ |F_e_ = 3, m_F_ = 3⟩, and the co-propagating and counter-propagating coupling fields, c_f_ and c_b_, drive the transition |F_g_ = 4, m_F_ = 2⟩ ↔ |F_e_ = 3, m_F_ = 3⟩ (see Figure 5). Considering the Doppler broadening effect caused by the thermal motion of atoms [22,34], the complex susceptibility of intracavity atoms to p_f_ light can be expressed as

(1) $$\chi_{3}=\frac{N_{0}\mu_{43}^{2}}{\varepsilon_{0}\hbar }\int_{-\infty }^{\infty }\frac{f(v)}{\left(\Delta_{p}^{\prime }+i\gamma_{1}\right)-\frac{\Omega_{cf}\left(\Omega_{cb}^{*}+\Omega_{cb}^{*}X_{1}\right)}{\left(\Delta_{p}^{\prime }-\Delta_{c}^{\prime }\right)+i\gamma_{2}}-\frac{\Omega_{cb}\left(\Omega_{cb}^{*}+\Omega_{cf}^{*}Y\right)}{\left(\Delta_{p}^{\prime }-\Delta_{c}^{\prime \prime }\right)+i\gamma_{2}}}dv,$$

where N_0_ is the atomic density at the given temperature (T), μ_43_ is the dipole moment matrix element of the transition |F_g_ = 4, m_F_ = 4⟩ ↔ |F_e_ = 3, m_F_ = 3⟩, ε_0_ is the vacuum permittivity, ħ is the reduced Planck constant, and f(v) = (m/2πk_B_T)exp(−mv^2^/2k_B_T) is the Maxwell velocity distribution (where v is the atomic velocity, m the atomic mass, and k_B_ the Boltzmann constant). $\Delta_{p}^{\prime }$ = Δ_p_ − ω_p_v/c, $\Delta_{c}^{\prime }$ = Δ_c_ − ω_c_v/c, and $\Delta_{c}^{\prime \prime }$ = Δ_c_ + ω_c_v/c. Ω_cf(b)_ is the Rabi frequency of c_f(b)_, γ_1_ is the decay rate between the excited state *|*F_e_ = 3,m_F_ = 3⟩ and the ground state |F_g_ = 4, m_F_ = 4(2)⟩, γ_2_ is the dephasing rate between two Zeeman sublevels of the ground state, and c is the vacuum light speed. X and Y are the scaling and circulator factors, which are expressed by the continued fraction (for the detailed expression and calculation process, see Ref. [34]).

The cavity transmission intensity (S) of the atom–cavity system normalized to the probe light is given by [36]

(2) $$S=\frac{{\left(1-R\right)}^{2}\gamma_{c}\exp(-\alpha l)}{{\left[1-R\gamma_{c}\exp(-\alpha l)\right]}^{2}+4R\gamma_{c}\exp(-\alpha l)\sin^{2}(\Phi /2)},$$

where R is the round-trip reflectivity of the cavity mirrors, αl = ω_p_lIm(χ)/c is the absorption depth (optical depth) of the intracavity atoms with length l, and γ_c_ is the intracavity linear loss. Φ = βl + ψ is the total cavity mode round-trip phase shift, with βl = ω_p_lRe(χ)/2c being the nonlinear phase shift resulting from the dispersion of the intracavity atoms and ψ = 2π(Δ_p_ − Δ_q_)/Δ_FSR_ being the linear phase shift in the cavity, where ∆_FSR_ = c/l is the free spectral range. Therefore, cavity transmission is critically dependent on the susceptibility of the intracavity atoms.

**Figure 6:**
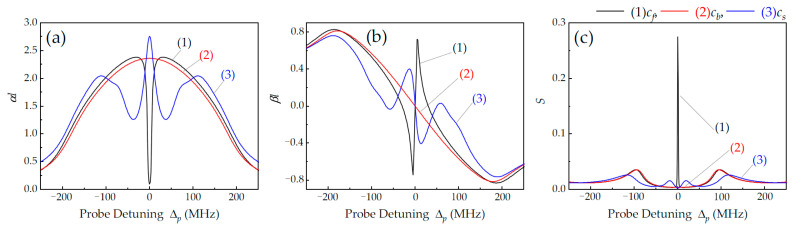
Theoretically calculated absorption depth αl (**a**), nonlinear phase shift βl (**b**), and cavity transmission S (**c**) versus probe detuning for different configurations of coupling fields: (1) only c_f_ with Ω_cf_ = 2π × 40 MHz, (2) only c_b_ with Ω_cb_ = 2π × 40 MHz, and (3) SW c_s_ with Ω_cs_ = 2π × 40 MHz. The main atomic parameters are γ_1_ = 2π × 4.6 MHz, γ_2_ ≈ 2π × 0.001 MHz, and N_0_ ≈ 3.0 × 10^15^ m^−3^ (T = 40 °C). The cavity parameters are R ≈ 0.94, Δ_FSR_ ≈ 566 MHz, and Δ_q_ = Δ_c_ = 0.

We theoretically simulated the absorption depth (imaginary part of χ) and the nonlinear phase shift (real part of χ) of the forward probe mode single passing through the intracavity atoms, as well as the normalized cavity transmission under different coupling field configurations, as shown in Figure 6. When only co-propagating c_f_ was injected, the intracavity atoms showed reduced absorption and sharp normal dispersion at Δ_p_ = Δ_c_ = 0, as shown by the black curves (1) in Figure 6a and 6b, respectively. Further, apart from two symmetric VRS peaks, a single-dark-state peak with narrowed linewidth appeared at atomic resonance, as shown by the black curve (1) in Figure 6c. In the configuration of the SW coupling field c_s_, we observed strong absorption (i.e., EIA) and steep anomalous dispersion, as shown by the blue curves (3) in Figure 6a and 6b, respectively. At the same time, the cavity showed strong absorption at Δ_p_ = Δ_c_ = 0, and two symmetrical double-dark-state peaks appeared near atomic resonance, as shown by the blue curve (3) in Figure 6c. The theoretical simulation of the single-to-double-dark-state transition is in good agreement with the experimental results in Figure 3a.

When only counter-propagating c_b_ was used, however, the atoms showed Doppler absorption and anomalous dispersion, as shown by the red curves (2) in Figure 6a and 6b, respectively. Additionally, due to the Doppler shift of the hot atoms, intracavity EIT disappeared at Δ_p_ = Δ_c_ = 0, with only VRS occurring on both sides of the atomic resonance, as shown by the red curve (2) in Figure 6c. The frequency separation of VRS (i.e., the cavity–atom coupling strength) is proportional to (g^2^N + Ω_c_^2^/4)^1/2^ [36,38], where g is the single-photon coupling rate for probe light and N is the number of involved intracavity atoms. The strong coupling between probe cavity mode and intracavity atoms induces large VRS (see the red curve (2) in Figure 6c), which significantly suppresses the transmission of probe light counter-propagating with respect to the coupling field. It is helpful to induce the generation of ONR by using EIT [18]. The theoretical simulation is inconsistent with the experimental results in Figure 3. The reason is that the strong-coupling effect of intracavity three-level atoms is not exactly the same as that of degenerate two-level atoms.

In a degenerate two-level atom–cavity system, coupling light and probe light drive the same transition. By ignoring the impact of the magnetic field (magnetic shielding material was used in our experiment), we can simplify the degenerate two-level system to a pure two-level system (see Figure 1b). In the configuration with only counter-propagating coupling light c_b_, with Ω_cb_ >> Ω_p_, the susceptibility of probe light p_f_ can be expressed as [39]

(3) $$\chi_{2}=\int_{-\infty }^{\infty }\left\{\left(1-\frac{\Omega_{cb}^{2}/\gamma_{1}^{2}}{1+{\Delta^{\prime }}_{p}^{2}+\Omega_{cb}^{2}/\gamma_{1}^{2}}\right)\frac{\left(\gamma_{1}-i\Delta_{c}^{\prime \prime }\right)\left[\gamma_{1}+i\left(\Delta_{p}^{\prime }-\Delta_{c}^{\prime \prime }\right)\right]+i\Omega_{cb}^{2}\Delta_{c}^{\prime \prime }/2\left(\gamma_{1}+i\Delta_{p}^{\prime }\right)}{\left(\gamma_{1}-i\Delta_{c}^{\prime \prime }\right)\left[\gamma_{1}+i\left(\Delta_{p}^{\prime }-\Delta_{c}^{\prime \prime }\right)\right]\left[\gamma_{1}-i\left(\Delta_{p}^{\prime }+\Delta_{c}^{\prime \prime }\right)\right]+\Omega_{cb}^{2}\left(\gamma_{1}-i\Delta_{c}^{\prime \prime }\right)}\right\}f(v)dv$$

**Figure 7:**
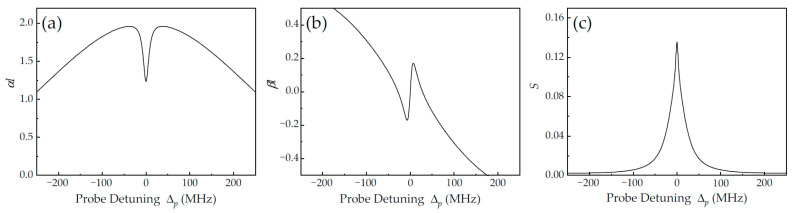
Theoretically simulated absorption depth αl (**a**), nonlinear phase shift βl (**b**), and cavity transmission S (**c**) of pure two-level atoms versus probe detuning in the configuration of a counter-propagating coupling field. The main theoretical parameters are the same as those in Figure 6.

By using Formulas (2) and (3), we plotted the theoretical simulation for a pure two-level atom system, as shown in Figure 7. Near atomic resonance, an absorption dip with linewidth broadening appears, accompanied by normal dispersion with a smaller slope, as shown in Figure 7a and 7b, respectively. It is worth noting that here, the “transparency” effect is quite different from the transparency caused by the quantum coherence of three-level atoms and is likely the “burning hole” effect. That is, strong-coupling light drives more atoms in the same ground state and reduces the effective atom number (N), which restrains the coupling strength of the atom–cavity system. Hence, a resonance transmission peak with linewidth broadening appears at Δ_p_ = Δ_c_ = 0 (see Figure 7c), which is in good agreement with the red curve (2) in Figure 3a. Although coupling light also plays a role in improving the cavity–atom coupling strength, there is competitive behavior. Clearly, weakening the coupling strength played a dominant role in our experiment. Therefore, based on the presence of intracavity EIT, reducing the optical power of the probe light is an effective means to achieve ONR. To set up a quantity simulation that perfectly matches the experimental results, rigorous and highly complex theoretical calculations that account for all Zeeman sublevels are necessary. 

**Figure 8:**
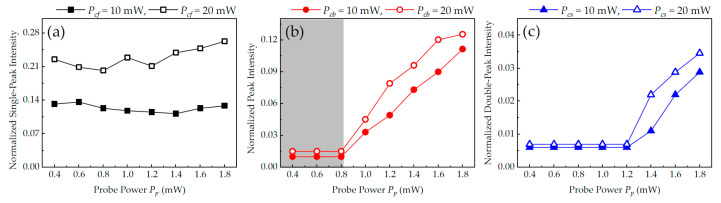
The normalized cavity transmission intensity of p_f_ versus the input optical power of the probe light in different coupling light configurations: (**a**) only c_f_, (**b**) only c_b_, and (**c**) SW c_s_. The grey area represents the range of ONR in the atom–cavity system. The other experimental parameters are the same as in Figure 3.

Based on the experiments, Figure 8 shows the normalized cavity transmission intensity as a function of the optical power of the probe light in the three different coupling light configurations. When only co-propagating c_f_ was injected, the normalized intensity of the single-dark-state peak had only slight fluctuations with the change in probe power. With the increase in the optical power of the coupling light, however, the intensity of intracavity EIT increased significantly. For example, the average intensity of the single-dark-state peak increased from 0.12 to 0.23 when the optical power of c_f_ increased from 10 mW to 20 mW, as shown by the solid and hollow squares in Figure 8a. For the case considering only counter-propagating c_b_, when P_p_ ≤ 0.8 mW, there was almost no transmission of the probe light from the cavity due to the strong-coupling effect of the atom–cavity system. That is, ONR based on intracavity EIT was achieved in this range, as shown by the gray area in Figure 8b. When P_p_ > 0.8 mW, cavity transmission without VRS suppressed the ONR effect, and the transmission intensity increased approximately linearly with the optical power of the probe light, as shown by the red circles in Figure 8b. As for the configuration of the SW coupling field, due to the lower transmission efficiency and larger intracavity loss, the double-dark-state peaks do not appear until probe power P_p_ > 1.2 mW, as shown by the blue triangles in Figure 8c. Similar results were also obtained for backward p_b_ and are not repeated here.

**Figure 9:**
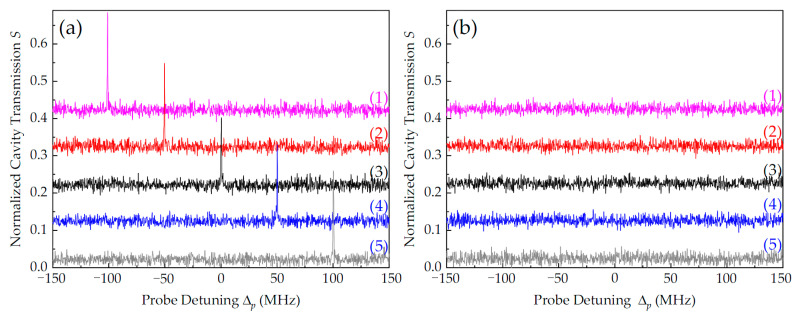
Normalized cavity transmission spectra of p_f_ under various coupling detuning values. Configuration considering (**a**) only c_f_ and (**b**) only c_b_. (1) Δ_c_ = −100 MHz, (2) Δ_c_ = −50 MHz, (3) Δ_c_ = 0, (4) Δ_c_ = 50 MHz, and (5) Δ_c_ = 100 MHz. The other experimental parameters are the same as those in Figure 3.

Additionally, the dependence of the ONR effect on coupling field detuning was experimentally investigated. Figure 9 presents the cavity transmission of p_f_ at different coupling detuning values Δ_c_. For the configuration considering only co-propagating c_f_ being used, the intracavity EIT peak shifted with Δ_c_, as shown in Figure 9a. Notably, cavity mode detuning, Δ_q_, hardly changed the frequency position, but it affected the intensity of intracavity EIT. The optimal transmission output occurred at Δ_q_ = Δ_c_. For the configuration considering only counter-propagating c_b_, no transmission (including VRS) was detected, as shown in Figure 9b. In our experiment, ONR transmission with high contrast (up to 90%) and narrowed linewidth (~0.6 MHz) was obtained when coupling detuning was in the range of −150 MHz < Δ_c_ < 150 MHz. For large coupling detuning values, the intracavity EIT signal still existed, but the transparency efficiency was very weak due to far-off atomic resonance, which reduced the contrast of ONR.

## 4. Conclusions

In conclusion, by using a degenerate two-level atom–cavity system, we experimentally obtained ONR based on intracavity EIT. For the degenerate transition F_g_ ↔ F_e_ (with F_g_ > F_e_), a linewidth-narrowed single-dark-state peak appears when the probe cavity mode co-propagates with the coupling field (absence of the Doppler effect), which results from the stable Λ-type coherence chains of all the Zeeman sublevels of the ground state. This is similar to the result for the three-level atom–cavity system. Differently, when the two beams counter-propagate, the coupling beam with the largest Rabi frequency can trap parts of the atomic population in the same ground state due to frequency degeneracy, which reduces the effective number of atoms that interact with the cavity mode and weakens the coupling strength of the atom–cavity system. Therefore, ONR based on intracavity EIT can only be established at a very weak probe intensity. This study’s findings further enrich the physical application of ONR and can also be applied in quantum information processing. Furthermore, due to the frequency degeneracy of probe light and coupling light, only one laser is needed to implement our experimental scheme, which could indeed be highly useful in the design of integrated optical quantum devices, such as optical switches and routers, and logical gate manipulation.

## Data Availability

Data are contained within the article.
